# Supplementary material for: Building flexible and robust analysis frameworks for molecular subtyping of cancers
Source: Mol Oncol. 2024 Jan 7;18(3):606–19. doi: 10.1002/1878-0261.13580 (PMC10920087; doi:10.1002/1878-0261.13580)
Supplement: Supplementary file 1 — Fig. S1. Purity estimates for CIT reference data. Fig. S2. Purity estimates for TCGA data. Fig. S3. Heatmaps of sample correlation to tissues. Fig. S4. Combat batch correction on five subtypes. Fig. S5. PCA of the four differently classified use case samples projected into CIT reference space. Fig. S6. Heatmaps of distances to different centroids. Table S1. Example patient characteristics. Table S2. Precision, recall, and weighted accuracy from different classification methods. Table S3. Distances to centroids for the four differently classified use case samples. [file MOL2-18-606-s001.pdf]

Supplementary materials

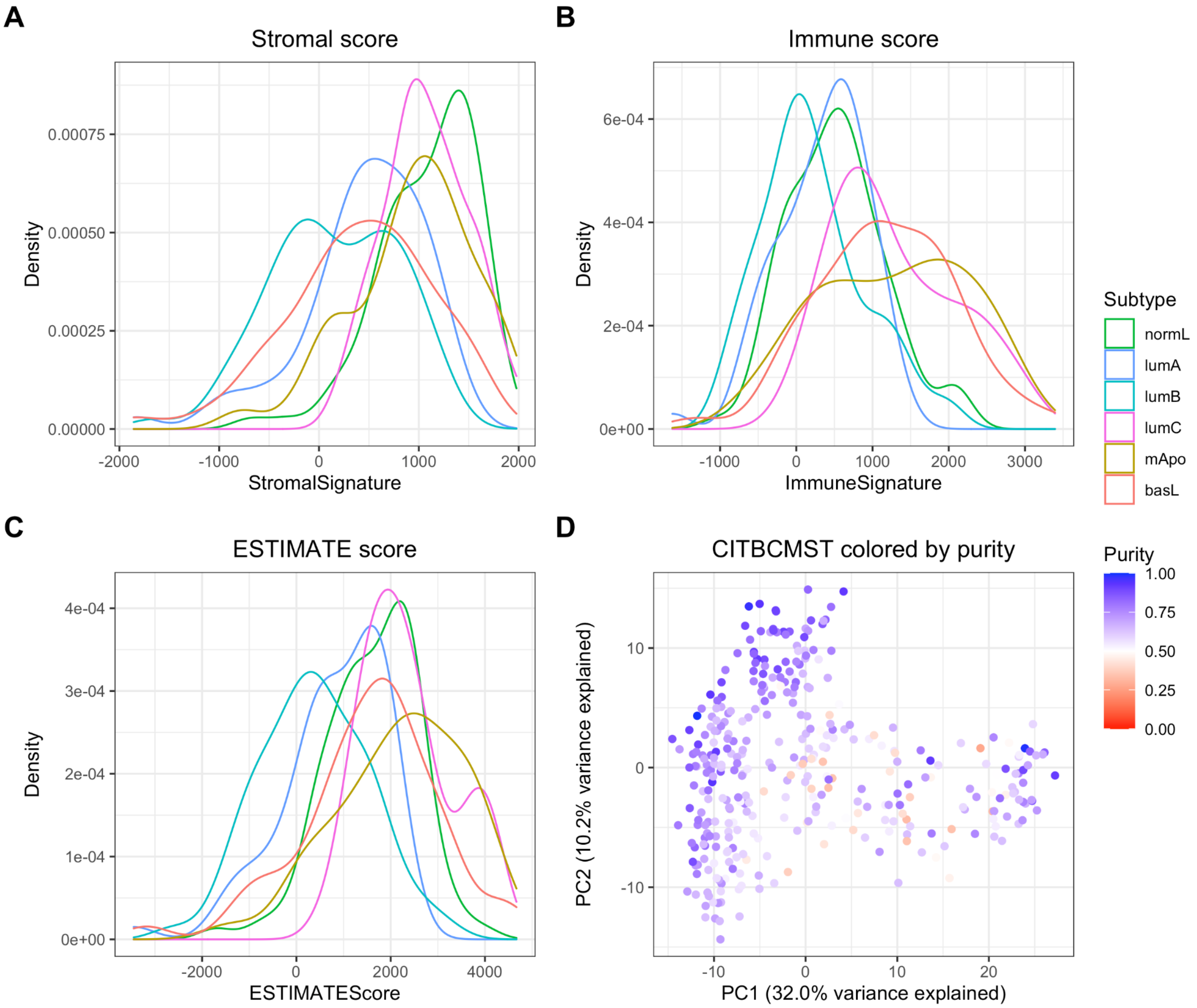

Supplementary Figure 1: Purity estimates for the CITBCMST training data. A) Stromal score distribution for each of the six subtypes. B) Immune score distribution for each of the six subtypes. C) ESTIMATE score distribution for each of the six subtypes. D) PCA plot of ranked CITBCMST training data colored by estimated tumor purity.

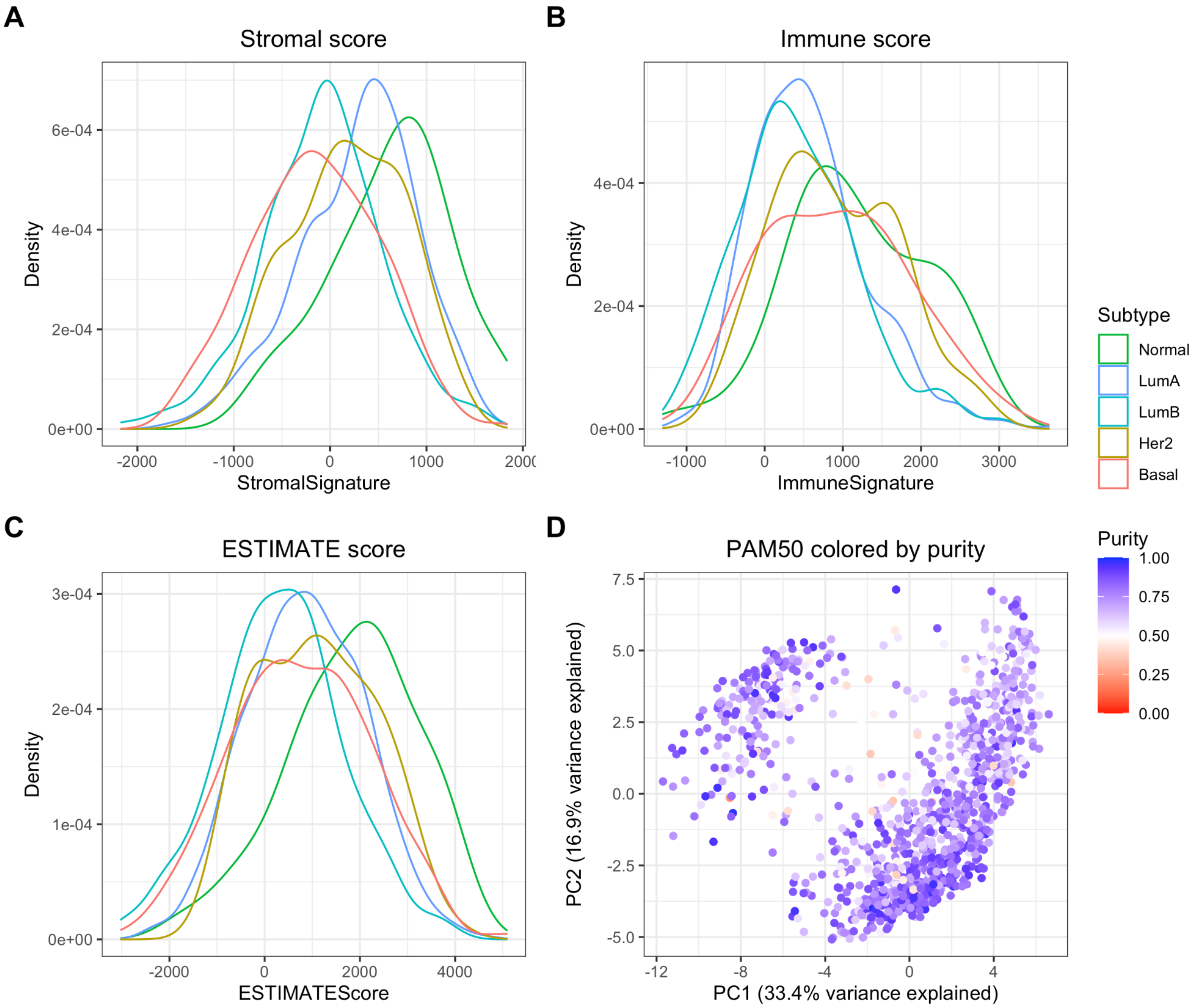

Supplementary Figure 2: Purity estimates for the TCGA data. A) Stromal score distribution for each of the six subtypes. B) Immune score distribution for each of the six subtypes. C) ESTIMATE score distribution for each of the six subtypes. D) PCA plot of ranked TCGA data colored by estimated tumor purity.

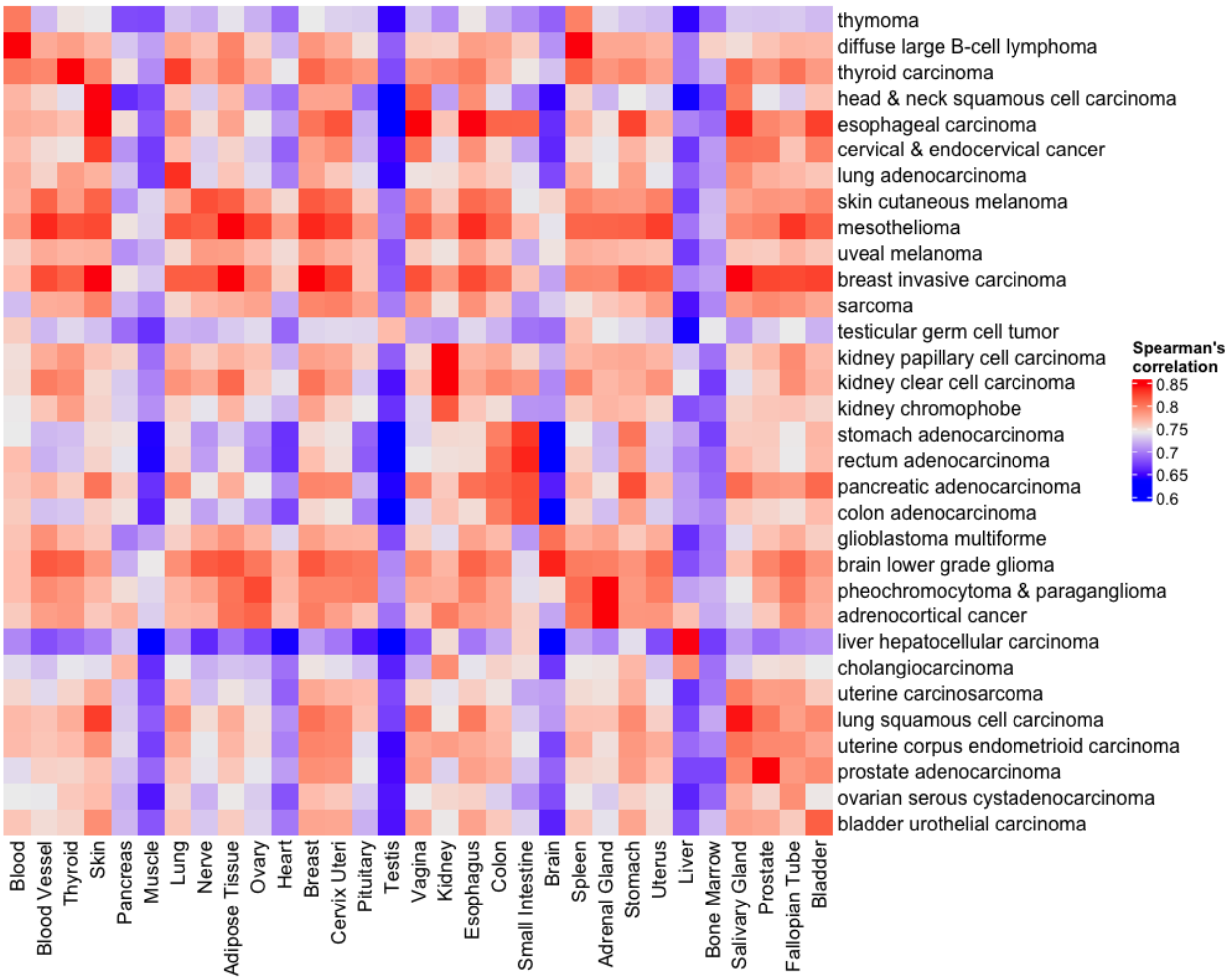
thymoma
diffuse large B-cell lymphoma
thyroid carcinoma
head & neck squamous cell carcinoma
esophageal carcinoma
cervical & endocervical cancer
lung adenocarcinoma
skin cutaneous melanoma
mesothelioma
uveal melanoma
breast invasive carcinoma
sarcoma
testicular germ cell tumor
kidney papillary cell carcinoma
kidney clear cell carcinoma
kidney chromophobe
stomach adenocarcinoma
rectum adenocarcinoma
pancreatic adenocarcinoma
colon adenocarcinoma
glioblastoma multiforme
brain lower grade glioma
pheochromocytoma & paraganglioma
adrenocortical cancer
liver hepatocellular carcinoma
cholangiocarcinoma
uterine carcinosarcoma
lung squamous cell carcinoma
uterine corpus endometrioid carcinoma
prostate adenocarcinoma
ovarian serous cystadenocarcinoma
bladder urothelial carcinoma
Blood
Blood Vessel
Thyroid
Skin
Pancreas
Muscle
Lung
Nerve
Adipose Tissue
Ovary
Heart
Breast
Cervix Uteri
Pituitary
Testis
Vagina
Kidney
Esophagus
Colon
Small Intestine
Brain
Spleen
Adrenal Gland
Stomach
Uterus
Liver
Bone Marrow
Salivary Gland
Prostate
Fallopian Tube
Bladder
Spearman's correlation
0.85
0.8
0.75
0.7
0.65
0.6

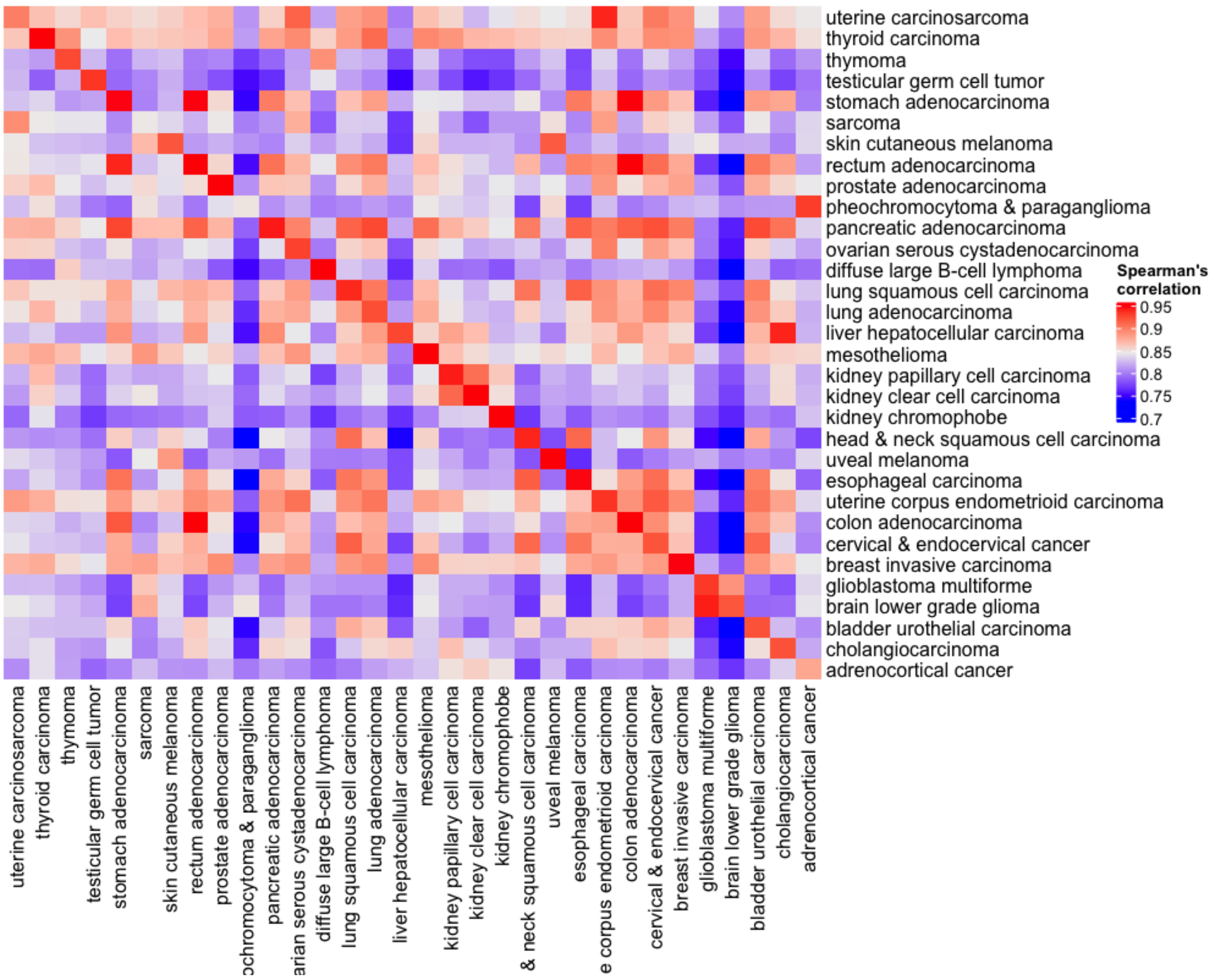

Supplementary Figure 3: Heatmaps of Spearman’s correlations from samples to tissue mean-based centroids in data sets of comprehensive tissue-specific expression profiles. One sample of each tissue type has been chosen to validate outlier detection. A) Heatmap of example samples vs. centroids defined from a collection of healthy tissues from the GTEx project. B) Heatmap of example samples vs. centroids from a collection of cancer tissues from the TCGA project.

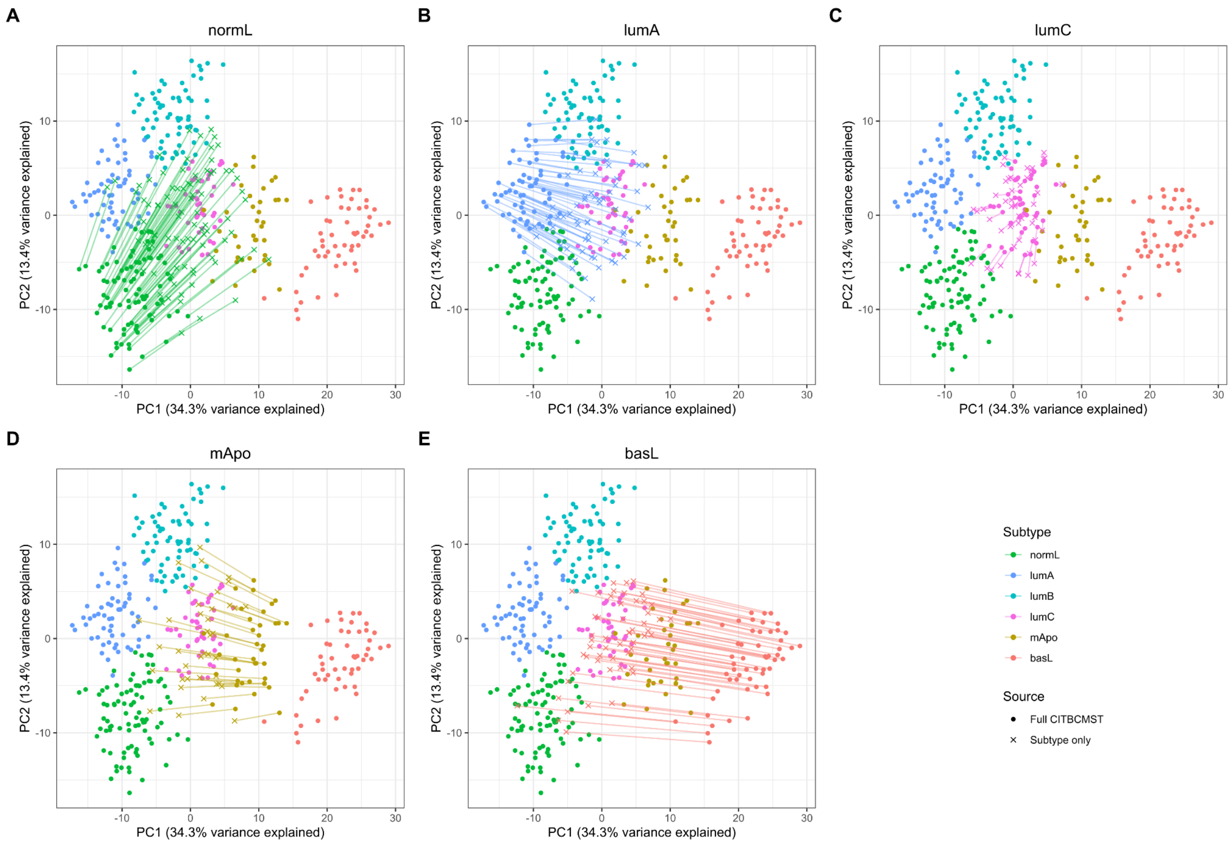

Supplementary Figure 4: ComBat on five subtypes individually and how it affects the location in PC space. Batch correction of the entire CITBCMST training set against the samples from each subtype alone, using ComBat with the full set as reference. Results for lumB are shown in Figure 5. A) normL, B) lumA, C) lumC, D) mApo, E) basL.

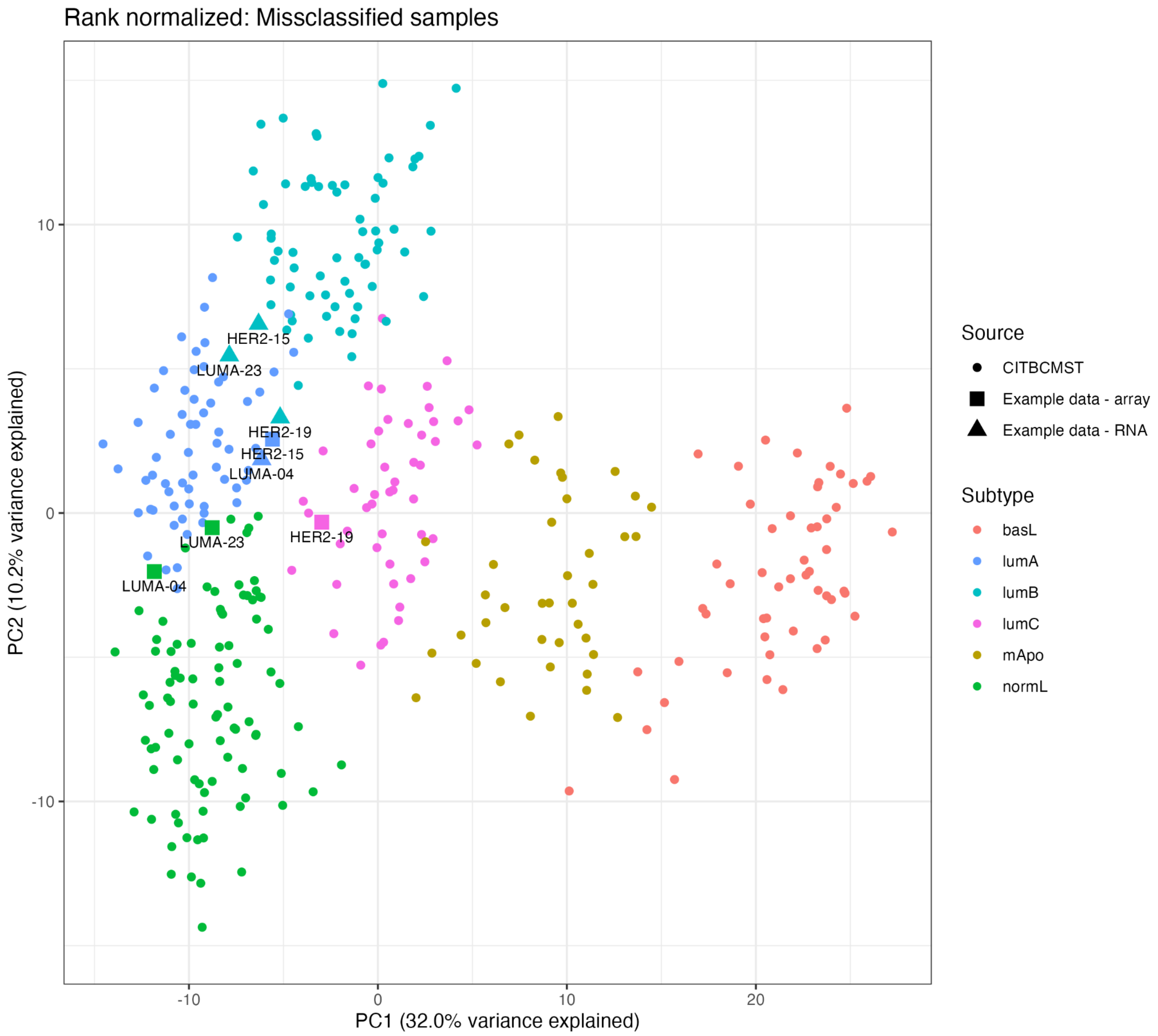

Supplementary Figure 5: PCA of the four differently classified use case samples projected into CIT reference space.

A)

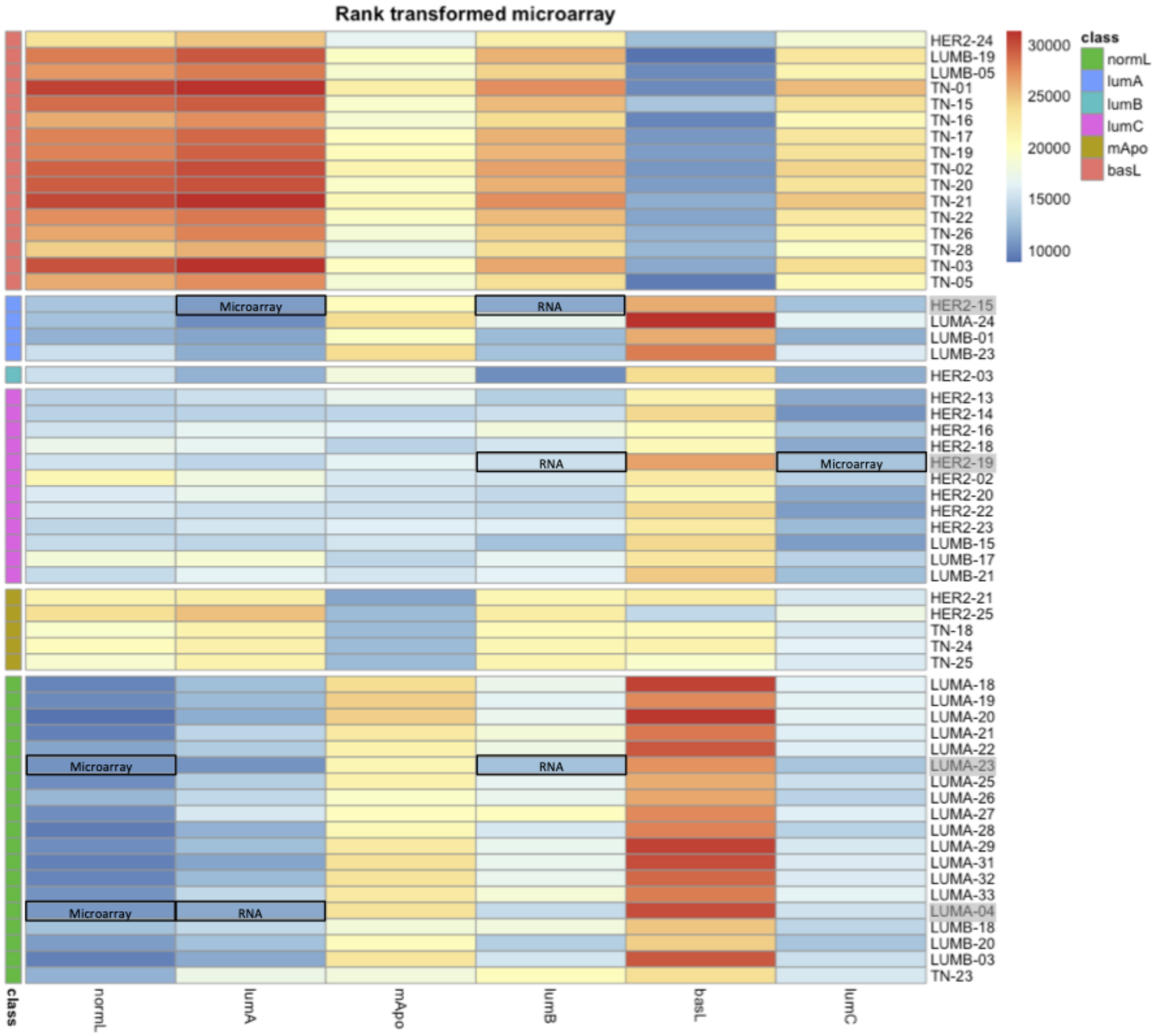

Rank transformed microarray
HER2-24
LUMB-19
LUMB-05
TN-01
TN-15
TN-16
TN-17
TN-19
TN-02
TN-20
TN-21
TN-22
TN-26
TN-28
TN-03
TN-05
Microarray
RNA
HER2-15
LUMA-24
LUMB-01
LUMB-23
HER2-03
HER2-13
HER2-14
HER2-16
HER2-18
RNA
Microarray
HER2-19
HER2-02
HER2-20
HER2-22
HER2-23
LUMB-15
LUMB-17
LUMB-21
HER2-21
HER2-25
TN-18
TN-24
TN-25
LUMA-18
LUMA-19
LUMA-20
LUMA-21
LUMA-22
Microarray
RNA
LUMA-23
LUMA-25
LUMA-26
LUMA-27
LUMA-28
LUMA-29
LUMA-31
LUMA-32
LUMA-33
Microarray
RNA
LUMA-04
LUMB-18
LUMB-20
LUMB-03
TN-23
class
normL
lumA
mApo
lumB
basL
lumC
30000
25000
20000
15000
10000
class
normL
lumA
lumB
lumC
mApo
basL

B)

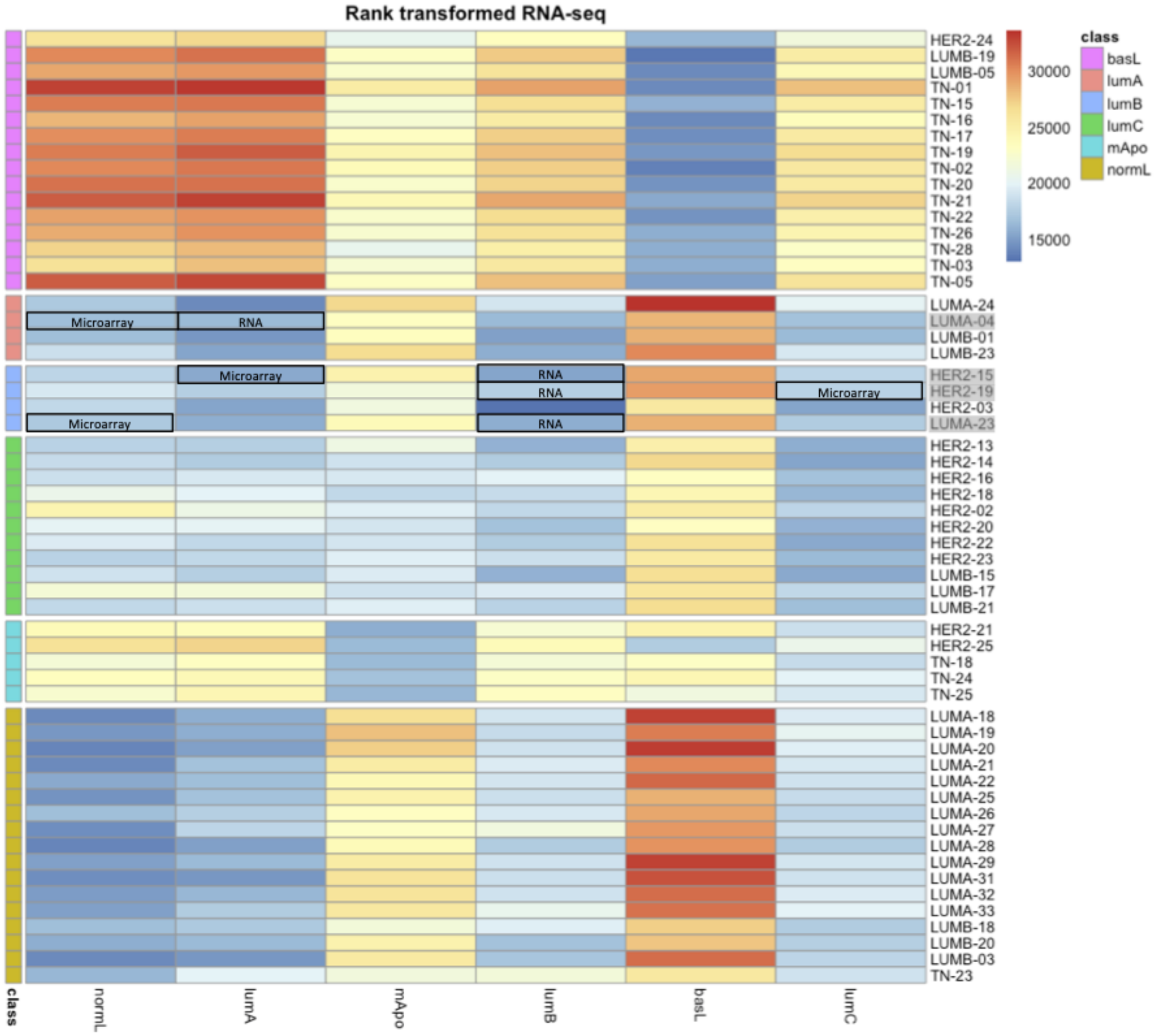

Supplementary Figure 6: Heatmaps of distances to different centroids for both microarray and RNA use case data. The four differently classified samples have been highlighted by black boxes in both heatmaps. The color indicates distance to centroids. A) Heatmaps for Array data. B) Heatmap for RNA-seq data.

Supplementary Table 1: Example patient characteristics.

Sample	Original name	ER status	PR status	HER2 status	Ki67 %	Grade
Sample 1	HER2-03	Positive	Positive	Positive	20	3
Sample 2	HER2-21	Negative	Negative	Positive	70	3
Sample 3	LUMA-18	Positive	Positive	Negative	5	1
Sample 4	LUMA-24	Positive	Positive	Negative	10	1
Sample 5	LUMA-27	Positive	Positive	Negative	5	1
Sample 6	LUMA-29	Positive	Positive	Negative	10	1
Sample 7	LUMB-01	Positive	Positive	Negative	30	3
Sample 8	LUMB-17	Positive	Negative	Negative	10	3
Sample 9	TN-18	Negative	Negative	Negative	30	2
Sample 10	TN-22	Negative	Negative	Negative	50	3

Supplementary Table 2: Precision, recall, and weighted accuracy from leave-one-out cross-validation of kNN, nearest centroid, and subtype signature functional class scoring for the PAM50-classification of the TCGA reference data. Highest precision per subtype is highlighted in green, highest recall is highlighted in blue, highest weighted accuracy is highlighted in purple.

	***k*-nearest neighbor**		**Distance-to-centroid**		**Subtype signature ssGSEA**	
Subtype	Precision	Recall	Precision	Recall	Precision	Recall
**Normal**	0.814	0.564	0.368	0.897	0.472	0.897
**LumA**	0.914	0.950	0.992	0.669	0.842	0.968
**LumB**	0.853	0.829	0.624	0.949	0.923	0.552
**Her2**	0.871	0.839	0.806	0.975	0.890	0.901
**Basal**	0.984	0.978	0.989	0.978	1	0.826
Weighted accuracy	0.832		0.894		0.829	

Supplementary Table 3: Distances to centroids for the four differently classified use case samples. A) Distances for the RNA-seq samples.. B) Distances for the microarray samples.

A)

RNA distances	normL	lumA	lumB	lumC	mApo	basL	Array class	RNA class
**HER2-15**	18033.5	15580.5	15442.5	17997	24514	28870	lumA	lumB
**HER2-19**	19320	17704	17645	17752	21426	28490.5	lumC	lumB
**LUMA-23**	17135	15782	15702	17485.5	23746	28490.5	normL	lumB
**LUMA-04**	16669.5	16316	16469.5	16889	23202	28377	normL	lumA

B)

Array distances	normL	lumA	lumB	lumC	mApo	basL	Array class	RNA class
**HER2-15**	13267.5	10869.5	11670.5	13003.5	20432.5	25877.5	lumA	lumB
**HER2-19**	15211	14321	14963	13239	16660	26405	lumC	lumB
**LUMA-23**	10454	10555	12999	13109	21006	27159	normL	lumB
**LUMA-04**	10670	11604	14728	15298	23115	30061.5	normL	lumA
